# Analysis of Formative and Evaluative Activities on Statistical Graphs in Textbooks for Chilean Rural Multigrade Education

**DOI:** 10.3390/ejihpe14050092

**Published:** 2024-05-15

**Authors:** Matías Bustamante-Valdés, Danilo Díaz-Levicoy, Eduardo Alarcón-Bustamante

**Affiliations:** 1 Programa de Doctorado en Didáctica de la Matemática, Universidad Católica del Maule, Talca 3460000, Chile; matias.bustamante.1267@alu.ucm.cl; 2 Facultad de Ciencias Básicas, Universidad Católica del Maule, Talca 3460000, Chile; 3 Millennium Nucleus on Intergenerational Mobility: From Modelling to Policy (MOVI), Santiago 7802436, Chile; esalarcon@uc.cl

**Keywords:** rural education, statistic, primary schools, statistics education, textbooks, statistical graphs, one teacher schools, data visualization

## Abstract

The aim of this paper is to analyze the formative and evaluative activities involving statistical graphs in the new textbooks for Chilean rural multigrade education. The methodology is qualitative, at a descriptive level and uses the content analysis technique. The sample is made up of the six primary education textbooks distributed by the Ministry of Education for rural multigrade schools. The results show the predominance of the bar chart, semiotic level 3, the task of calculating and the personal context in both types of activities, although with respect to the reading level, it is evident that level 4 predominates in the formative activities and level 2 in the evaluative ones. According to the results, it is recommended to incorporate graphs proposed by the curricular guidelines of the Ministry of Education, which are absent in textbooks as well as to include evaluative activities that require reflection on the nature of the data, context, representation and conclusions obtained from them.

## 1. Introduction

Currently, there is a growing interest in studying the processes of teaching and learning statistics in the early years of schooling. This interest is primarily due to the integration of statistics into the curriculum guidelines of various countries (e.g., [1,2,3,4,5]) and the need to educate statistically literate citizens. Specifically, it involves the development of skills in reading, interpreting, and critically evaluating information obtained from statistical representations [6]. Given that students have access to a wealth of data through the media, which are mostly presented in statistical graphs [7,8], these graphs are considered elements of statistical culture [6,9,10].

In the Chilean context, international studies have shown a low proficiency in statistics among primary education students [11,12], including their ability to work with statistical graphs, despite efforts to improve teaching. Many of these schools taking this assessment are located in rural areas [13], where a series of factors and characteristics, such as multigrade classrooms (with students of different ages together in the same classroom), contribute to lower achievement compared to urban schools [14]. While there have been research efforts in the field of mathematics education in rural settings [15,16,17,18,19,20], there are limited studies that address the current situation in the field of statistics and statistical graphs in these areas.

On the other hand, the role of textbooks is considered significant as a means to work with curriculum topics [21,22], as they offer a systematic and continuous approach to content [23], enabling the implementation of curriculum guidelines in the classroom [24,25]. Textbooks also help reduce the cultural gap [26]. Notably, textbooks propose formative activities for in-class work and others intended to assess learning at the end of a unit. Therefore, it is essential to consider the evaluation process, as it provides valuable information for both teachers and students [27], allowing the collection of qualitative and quantitative data to assess their achievements and compliance with curriculum guidelines’ objectives, thus facilitating teachers’ decision making.

In accordance with the above, the following research question arises: What are the characteristics of the formative and evaluative activities with statistical graphics in the textbooks proposed by the Chilean Ministry of Education for rural education? From this question, the general objective of this study emerges, which is to analyze the formative and evaluative activities that use statistical graphs in textbooks for rural schools with multigrade classrooms in order to know how the Chilean Ministry of Education proposes activities with statistical graphics for rural multigrade schools.

### 1.1. Rural Multigrade Schools and Statistical Graphs

The most important idea to consider when conducting a study in a rural school is undoubtedly the characteristic of multigrade classrooms [28]. Multigrade classrooms are understood as the natural way of teaching children of different ages together [29]. These rural multigrade schools typically have at least one combined classroom with students ranging from first to sixth grade [30]. Moreover, they tend to be the only schools in their locality and are usually located in communities with populations of no more than 500 inhabitants [31,32].

The teaching of statistics was integrated into primary education a decade ago [3]. The curriculum is organized into five thematic areas: (a) numbers and operations, (b) patterns and algebra, (c) geometry, (d) measurement, and (e) data and probability. The latter includes the study of statistics and specifies learning objectives related to statistical graphs aligned with the textbooks for rural education, which are different from those delivered in urban schools. Table 1 presents the learning objectives for multigrade rural education for each primary school grade. It shows that the use of these representations begins in first grade and continues through sixth grade, with the following types of statistical graphs specified: pictograms (first to fourth grade), bar (second to sixth grade), dot plots (third and sixth grade), line charts (fifth grade), stem-and-leaf plots (fifth and sixth grade), and pie charts (sixth grade).

### 1.2. Reading Levels

The interpretation of statistical graphs is a complex task that involves various mathematical and statistical concepts, making its study quite interesting. Among the contributions in this field, the work carried out by Curcio and collaborators [34,35,36] stands out. They propose and describe different levels of sophistication when it comes to reading a statistical graph (see Table 2).

### 1.3. Levels of Semiotic Complexity

Another process that involves a variety of mathematical and statistical elements is the construction of statistical graphs. That is why Arteaga and collaborators [7,37] describe levels of semiotic complexity (see Table 3).

### 1.4. Background on Activity Analysis with Statistical Graphs in Textbooks

Currently, the analysis of textbooks has become a well-established research area within the fields of Mathematics Education [38] and Statistics Education [39,40].

Internationally, primary school textbooks have been compared, considering different mathematical concepts. For example, fourth grade primary textbooks from Indonesia and Singapore are compared in angle topics [41]. Their results show that Indonesian textbooks provide greater learning opportunities than Singaporean textbooks, although the latter are dominated by visual form, as opposed to the former which contains a purely mathematical form. Furthermore, in the United States, the topic of patterns in K-12 textbooks has been analyzed [42], stating that most activities are simplistic and there is little variability in complexity in the materials and structure of the pattern. It is necessary to optimize the tasks to provide more learning opportunities for students. Also, problem-solving activities are analyzed considering computational thinking among Chinese and Canadian textbooks [43], using computational thinking tools broken down into three steps: understanding the problem, designing and carrying out plans, and looking back. The presence of computational thinking is evident in the textbooks of both countries and they require their students to make generalizations from their knowledge.

On the other hand, text analysis has been carried out, where activities with statistical graphs are analyzed. It is essential to mention one of the earliest studies in Spanish [44], where a series of textbooks for primary education were analyzed. The units of analysis in this study included the type of graph and the required task. The results helped identify various types of statistical graphs, such as bar charts, pictograms, Cartesian coordinate diagrams, line charts, histograms, population pyramids, and pie charts. Additionally, various tasks were identified, including reading, completing, interpreting, constructing, creating graphs, and writing coordinates, among others.

Another significant study was conducted by [45], who used different units of analysis to investigate textbooks in a comparative study between Spain and Chile. These units included the type of graph, type of task, reading level, and semiotic complexity level. They used these units to analyze textbooks from various countries, including Argentina [46], Peru [47], and Costa Rica [48]. Their results showed that bar charts and reading level 2 were predominant in all countries. Concerning the tasks, calculating was the most frequent task in Chile, Argentina, and Peru, while reading was prominent in Spain, and both reading and calculating were common in Costa Rica. Regarding semiotic complexity, level 2 was prevalent in Argentina and Costa Rica, while level 3 was more common in Chile, Spain, and Peru.

In Chile, especially in textbooks for multigrade rural education, [49] analyzed activities using units of analysis such as the type of graph, reading level, semiotic complexity level, type of task, and data context. Their results indicated the dominance of bar charts, reading level 2, semiotic complexity level 3, calculating tasks, and personal context, respectively. These results were later confirmed by [50] in their evaluation of activities in textbooks for multigrade rural education in Chile.

In summary, international research on textbook analysis with statistical graphics shows the predominance of the bar graph, reading level 2, semiotic complexity level 3, and the task of calculating, although, occasionally, in some countries, the reading task and semiotic level 2 predominate.

In 2021, new textbooks were published for multigrade rural education in Chile. However, according to previous research, there is no evidence of studies that analyze and compare these new textbooks with the previous ones [33] used in this context.

## 2. Materials and Methods

This study is of a qualitative nature [51], at a descriptive level [52], and utilizes the content analysis method [53]. The sample is purposive and consists of the textbooks recommended for use in 1st to 6th grade multigrade rural primary education courses in Chile, as presented in Table 4. In the same way, it is necessary to mention that within the MINEDUC textbooks, there are activities with different purposes, in the first instance there are activities to work class by class with a formative purpose and, in the second instance, there are activities at the end of the unit, which are used to evaluate learning.

In this study, the following units of analysis were considered, which allow us to characterize the activities with statistical graphs in an exhaustive manner and are the most relevant to achieving our research objective:

- Type of graph: These are the ones specified in the curriculum guidelines of [33].
- Reading level: These are described by Curcio and collaborators [34,35,36], which include (a) reading the data, (b) reading within the data, (c) reading beyond the data, and (d) reading behind the data.
- Semiotic complexity level: These are proposed by Arteaga and collaborators [7,37].
- Type of task: These are described in previous research [39,49], which include tasks such as (a) reading, (b) calculating, (c) completing, (d) constructing, and (e) justifying, among others.
- Context: These are the contexts described in PISA [54], which are (a) personal, (b) work-related, (c) social, and (d) scientific.

For the analysis of the categories, all the activities in the textbooks that involved statistical graphics were searched. For each of them, the main author identified the reading level, level of semiotic complexity, types of tasks and context, being discussed and confirmed together with the other authors of the study. In addition, it should be noted that in the case of the reading level, having different questions in the same activity is considered the highest demanded by the activity.

In some units of analysis, such as type of graph, task, and semiotic level, it is possible to observe more than one category within the same activity because multiple charts may appear when conducting an activity. In such cases, all representations are counted, so the total value of the unit of analysis may be greater than the number of activities.

## 3. Results

Table 5 presents the distribution of formative and evaluative activities involving statistical graphs in textbooks for multigrade rural education from first to sixth grade of primary education. In the table, a total of 102 activities are shown, with 82 (80.4%) being formative activities and 20 (19.6%) being evaluative activities. Regarding formative activities, the highest frequency is in the sixth grade (31.7%), followed by the fifth grade (18.3%) and the first grade (17.1%). Additionally, there is a significant difference in the number of activities between the sixth and fourth grades. Concerning evaluative activities, the majority are in the fifth grade (25%), although the difference is not significant when compared to the other grades. When considering all activities, there is a predominance in the sixth grade (29.4%), followed by the fifth grade (16.7%) and the first grade (15.7%). According to the above, it can be observed that the textbooks propose a reduced number of evaluative activities, implying that it may not be possible to collect enough information to evaluate knowledge in order to subsequently make decisions in the teaching process.

### 3.1. Type of Graph

According to the types of graphs observed in the formative and evaluative activities analyzed, Table 6 shows the predominance of bar charts, corresponding to 34.9% and 40%, respectively. And, in second place, it is followed by the pictogram with 21% and 25%. In addition, there are formative activities with statistical graphs that do not appear in the evaluative activities, as is the case of the bar chart and dots in the fifth grade, and in the fourth grade, only the pictogram is observed in the evaluative activities. In this case, it is striking that there are courses that are evaluated through activities with graphs that are not proposed to work in a formative way (pictogram in fourth), and that also, the work with all the proposed graphs (bars and points in fifth) is not evaluated. Therefore, it is evident that there is a contradiction in the Chilean curricular guidelines regarding the use of pictograms, bar graphs and points, which may cause students to have confusions that lead them to make mistakes in data analysis and decision making.

The following is a description of reading levels, levels of semiotic complexity, task types and context types with examples based on activities from the textbooks analyzed.

### 3.2. Reading Levels

**Figure 1:**
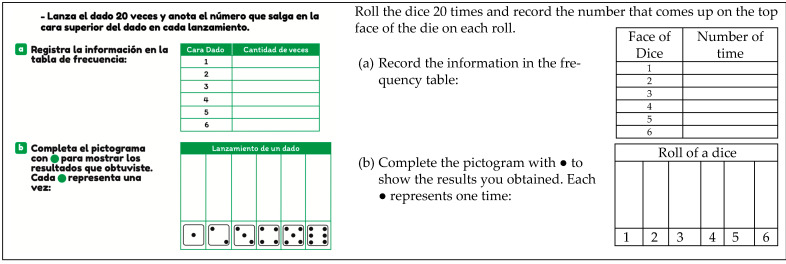
Example of reading level 1 (T1, p. 75).

In this section, we analyze the reading levels defined by Curcio and colleagues [32,33,34]. Reading level 1 is exemplified by Figure 1, where the student engages in literal readings of data. Specifically, the student must read data completed in the table by rolling a die 20 times and transferring them to a pictogram, where each icon represents a roll.

**Figure 2:**
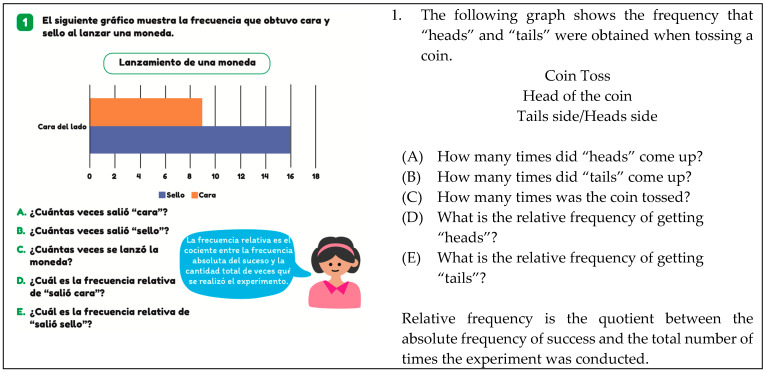
Example of level 2 reading (T6, p. 109).

An example of reading level 2 is illustrated in Figure 2, where the student is required to perform simple mathematical calculations to answer questions. In this case, it becomes evident in question C, as the student needs to add up frequencies to determine the number of times the coin was tossed.

**Figure 3:**
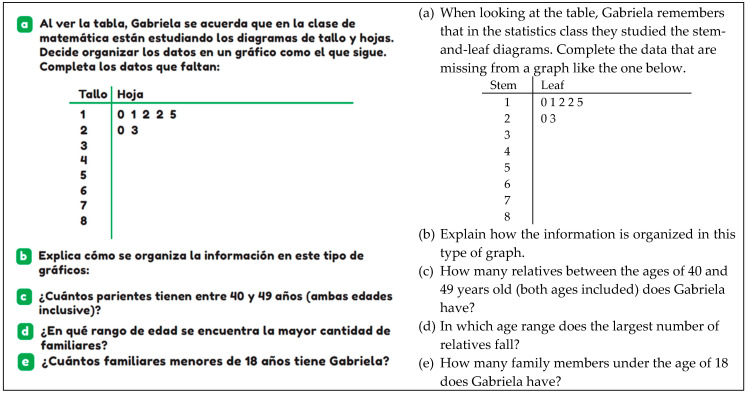
Example of reading level 4 (T5, p. 95).

An example of reading level 4 is illustrated in Figure 3, where the student must provide reasoning about how the information is organized in the stem-and-leaf plot. This comes after completing the plot with data provided about the ages of their family members.

Table 7 shows the predominance of reading level 4 in formative activities (68.3%) and reading level 2 (80%) in evaluative activities. It is also evident that reading level 4 is present in all primary education courses, but only in formative activities. It is necessary to point out the difference that exists between the reading levels required in the formative and evaluative activities. The latter are focused on algorithmic work, evaluating students in procedures in which they do not need to understand concepts related to the calculations, not allowing them to evaluate the way in which they obtain conclusions and the effectiveness in communication. On the other hand, there are no activities at reading level 3, in which it is required to make predictions from the data, implying that there is not a complete understanding of statistics, since this process is relevant to allow students to identify patterns in the data and to extrapolate them to future events, an important aspect to understand phenomena of everyday life related to statistics.

### 3.3. Levels of Semiotic Complexity

**Figure 4:**
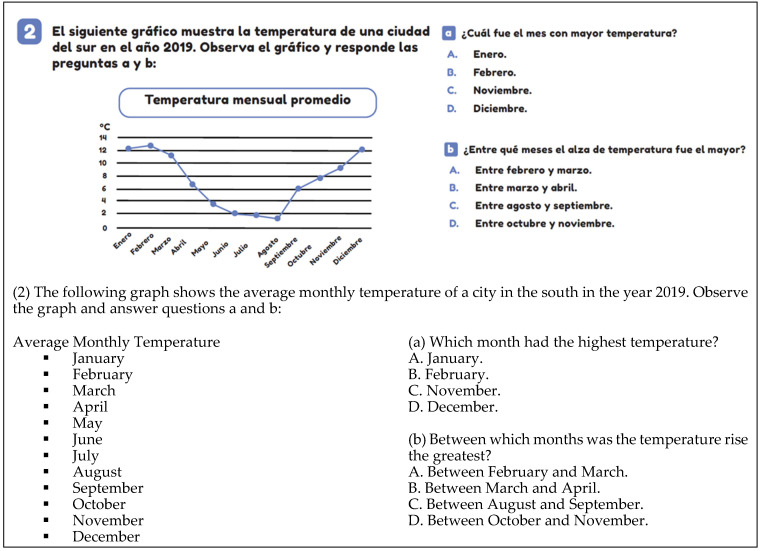
Example of level 2 of semiotic complexity (T5, p. 122).

An example of semiotic level 2 is illustrated in Figure 4, where the variation in temperature for a southern city in the year 2019 is shown. In this graph, temperature data are represented, but there is no frequency distribution. The concept of a variable is present, but there is no distribution of frequencies.

**Figure 5:**
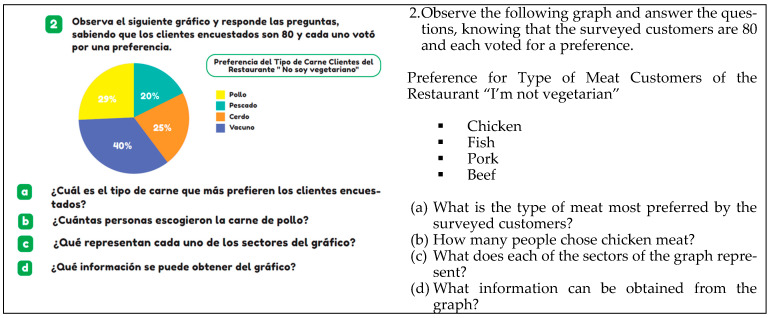
Example of level 3 of semiotic complexity, (T6, p. 85).

Semiotic level 3 is exemplified in Figure 5, which displays the percentage frequencies of distribution regarding customer preferences for types of meat at the “I’m not vegetarian” restaurant. In this activity, concepts of variables, frequency, and frequency distribution are involved.

**Figure 6:**
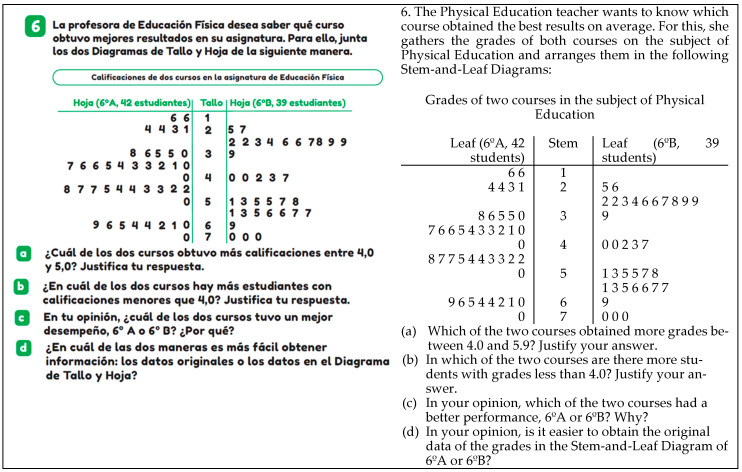
Example of level 4 of semiotic complexity (T6, p. 99).

Figure 6 exemplifies semiotic level 4, presenting in the stem-and-leaf plot distributions by class (sixth grade A and sixth grade B) of grades in the subject of Physical Education. In other words, two distributions are represented in a single statistical graph, allowing for a comparison of the behavior of both sets of data.

Among the levels of semiotic complexity in the analyzed activities, Table 8 shows the predominance of semiotic level 3 in both formative activities (79.5%) and evaluative activities (80%), appearing in all primary education courses. Conversely, the least frequent semiotic level in both types of activities is semiotic level 2, with 8.4% and 5%, respectively. Additionally, semiotic level 4 is only evident in the sixth grade course. In this aspect, by having this concentration at semiotic level 3, students may be limited by not having the opportunity to understand the characteristics of other data, such as temporal trends or patterns. Similarly, there are few activities where data are presented without the use of frequencies, so it is apparently not as relevant in elementary grades for students to visualize the relationship between two variables or data trends.

### 3.4. Task Type

In the reading task, students must engage in a literal reading of data or elements in the statistical graph, such as the title, axes, scale, and others. An example of this task is shown in Figure 1, where the question “How many times did ‘heads’ appear?” (question a) is asked.

The calculating task involves students performing calculations to obtain information from the graph. This may include comparing data or adding frequencies, among other calculations. This task is exemplified in Figure 2, where the question “What is the relative frequency of ‘heads’?” (question d) is asked.

The completing task occurs when students must finish constructing a statistical graph by assigning missing data, adding titles, labels, or elaborating bars, among other things. This type of task is exemplified in Figure 3, where, based on data about the ages of their family members, students must complete the stem-and-leaf plot.

**Figure 7:**
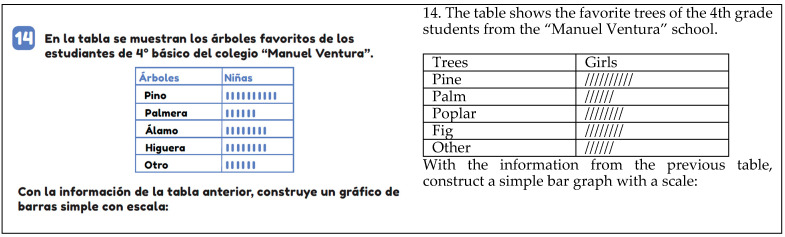
Example of a build task (T4, p. 104).

In the constructing task, students are required to create a graph based on data presented in a table or without the use of a table. They need to determine the scale, general and axis titles, labels, and other elements. This task is exemplified in Figure 7, where students are asked to construct a simple bar chart based on data from a table showing favorite trees (pine, palm, poplar, fig, other) of fourth grade students at Manuel Ventura School.

**Figure 8:**
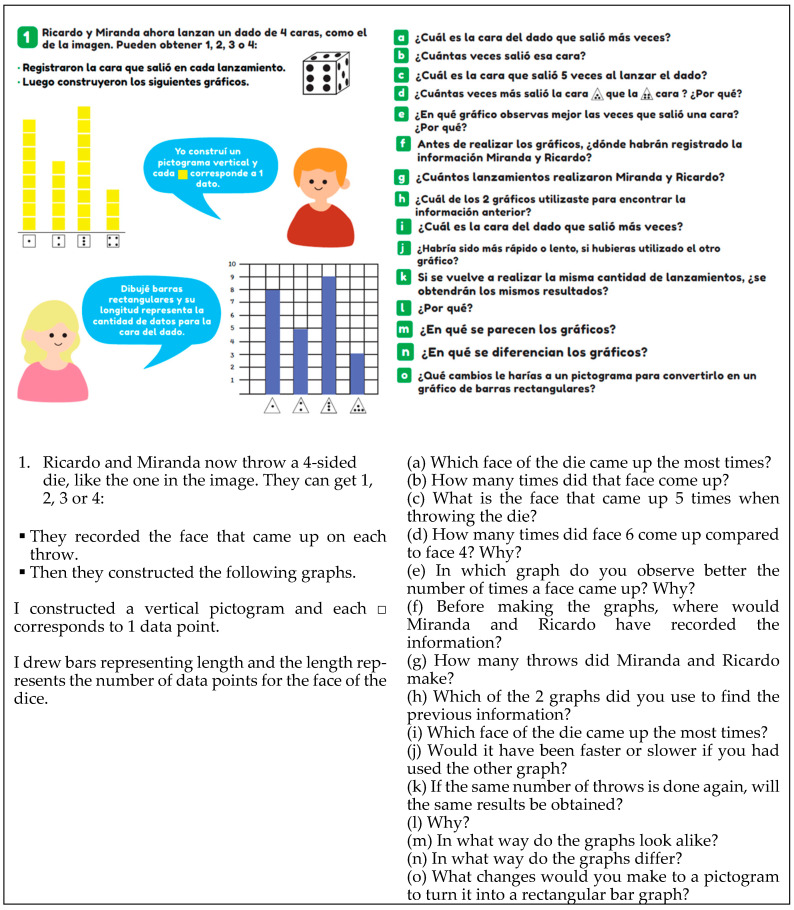
Example of a compare task (T2, p. 72).

Regarding the task of justifying, it involves students providing reasoning for certain situations, explaining processes, or making arguments based on their perspectives. Figure 8 exemplifies this when asking, “What changes would you make to the pictogram to transform it into a bar chart?” (question O).

**Figure 9:**
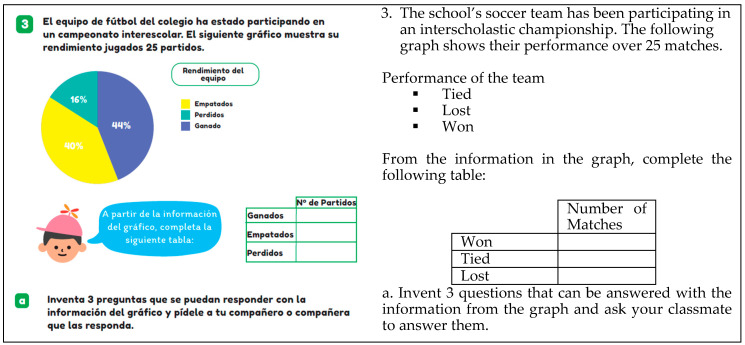
Example of a question invention task (T6, p. 86).

The task of creating questions involves students generating questions to obtain information from the data presented in a statistical graph. An example of this task is shown in Figure 9 because students are asked to invent three questions that can be answered using the data from the pie chart, which pertains to the team’s performance (games tied, lost, and won).

The task of comparing involves comparing two graphs to determine which one is more suitable for representing specific data based on their nature, which one shows the data more clearly, and so on. This type of task is evident in Figure 8, where the question “In which statistical graph is it easier to see the number of times ‘heads’ appeared?” (question e) is asked.

Regarding the tasks in the analyzed activities, Table 9 shows that the calculating task predominates in both formative and evaluative activities, with 66.6% and 85%, respectively. The justifying task appears only and in significant numbers in formative activities (57.8%). Additionally, the tasks of creating questions, comparing graphs, and converting to a table are not evident in evaluative activities. Furthermore, there is an inconsistency between formative and evaluative activities, as in all courses, there are tasks proposed for formative work that are not evaluated, especially tasks related to justifying, generating questions, comparing, and converting to a table.

### 3.5. Types of Contexts

The personal context is when the student is required to engage in an activity related to a situation close to them, their family, or peers. This context is identified in Figure 1, where a game scenario is presented (rolling a die 20 times), which they must record in a table.

The work context becomes evident when the activity is framed within the world of employment. For example, in Figure 5, the preferences of 80 restaurant customers regarding the type of meat they prefer (chicken, fish, pork, and beef) are presented.

**Figure 10:**
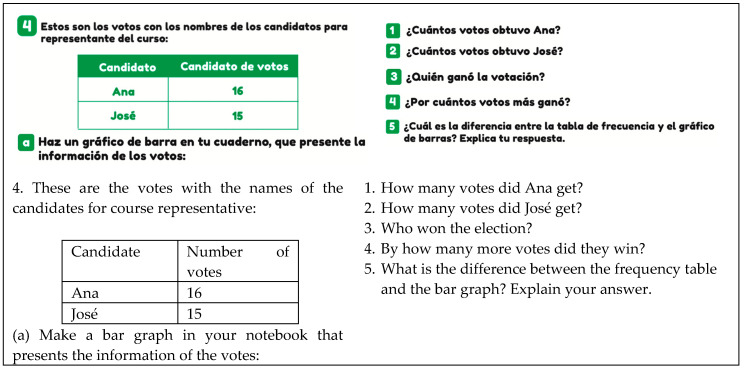
Example of social context (T2, p. 83).

The social context is considered when the theme involves democratic processes or is of local, regional, or national interest. Specifically, in Figure 10, a situation is presented where a vote is conducted with two candidates (Ana and José) to be the class representative, which must then be represented in a bar chart.

The scientific context arises when mathematics is applied in nature, science, technology, or within the discipline itself. This context is exemplified in Figure 4, where temperatures (°C) in a city in the year 2019 are presented. In this case, the scientific context becomes evident as a graphical representation is used to display data from nature.

Regarding the types of contexts identified in activities with statistical graphs, there is a predominance of the personal context, both in formative activities (61.5%) and evaluative ones (75%). In second place, the work context is present with 30.1% and 20%, respectively. Additionally, the social context is only observed in formative activities and at a very low frequency (see Table 10). Having most of the activities within the personal context of the students promotes the importance of the concepts discussed as they are related to everyday life. However, it can also present limitations by not exploring diverse situations or by not giving relevance to other contexts, as it can restrict the understanding of the real world—in this case, the social and scientific context.

## 4. Conclusions

When analyzing formative and evaluative activities with statistical graphs and considering the units of analysis, the following conclusions can be drawn:

In terms of the types of graphs used, there is a predominance of bar charts, similar to what was found in the work of [49] for formative activities and [50] for evaluative activities. Additionally, these results align with studies on activity analysis in textbooks for countries such as Argentina [46], Peru [47], and Costa Rica [48]. It is worth noting that in some courses, there is a lack of work with the statistical representations recommended by MINEDUC for teaching and assessment purposes, such as the absence of bar charts in the fifth grade course and pictograms in the fourth grade course. Therefore, it is recommended to integrate these types of representations to ensure consistency between MINEDUC guidelines and rural multigrade Chilean textbooks, implying that it is necessary to update the textbooks analyzed so that they do not generate confusion and hinder the learning of Chilean students.

Regarding reading levels, the predominant level is level 4, in contrast to [49], which focused on level 2 for formative activities. This implies that current rural education textbooks aim to encourage students to reflect on data collection, context, methods of representation, and the conclusions drawn, as opposed to older textbooks that emphasize algorithmic work. Interestingly, there is a lack of level 4 reading questions in evaluative activities, even though it is the most frequent level in formative activities. This absence is also observed in [50], indicating no significant changes in the way activities involving statistical graphs are evaluated in terms of reading levels. Level 2 reading activities are predominant, similar to [50], suggesting a lack of consistency in the way statistical graphs are taught and assessed from one class to another. Therefore, it is recommended to incorporate evaluative activities with a level 4 reading requirement, in order to develop critical thinking in students, focusing especially on decision making, allowing them to have a better understanding of statistical concepts and their link with everyday life. Finally, there is a lack of both formative and evaluative activities requiring a level 3 reading. This means that elementary school students do not make predictions based on data presented in statistical graphs as part of their learning process. Given its importance, it is recommended to incorporate this level into both types of activities (formative and assessment activities), those that include activities to identify patterns, trends, and others that require techniques to interpret data, which allows understanding real-world situations related to statistical graphics. In terms of semiotic complexity levels, level 3 predominates, similar to [49] for formative activities and [50] for evaluative activities in previous rural textbooks and international research [46,47,48]. This indicates that most graphs focus on data distribution. However, unlike previous textbooks [49,50], semiotic level 4 is used for stem-and-leaf plots and dot plots, implying the representation of more than one data distribution. This level was previously only evident in the double bar chart. In addition, it is worth mentioning the importance of graphs where frequencies are not presented and which appear scarcely in the textbooks analyzed, which allow us to work on the idea of variables, observe relationships between data, trends or patterns, which are relevant aspects to be able to interpret the data.

The most frequently required task is calculation, which aligns with the findings of [49,50]. This means that, in the analyzed activities, despite level 4 reading being the most common, simple calculations are notably included to obtain information within the various activities. Therefore, it is essential to develop evaluative activities that require different types of tasks, which allow students to have a better understanding of reading and construction of statistical graphs and, thus, to guide the teaching process.

Regarding the context of the data, personal context predominates, similar to [49]. However, there is a decrease in the inclusion of social context in the analyzed activities, with it even being absent in evaluative activities. Therefore, it is recommended to integrate this type of context, as it is essential for students to be aware of social situations in which they are involved, whether at a local, regional, or national level. Likewise, focusing on the situations in the context closest to the students is beneficial for their learning. However, it is necessary to balance the variation of the other contexts, allowing an integral development and increasing their understanding of reality given the applicability of statistics in the different areas of knowledge.

Based on the above study, it is suggested that a future projection could involve comparing formative and evaluative activities between rural and urban schools using these units of analysis to identify differences and similarities, with the purpose of evidencing if the textbooks and curricular guidelines consider the reality of each type of school in an adequate manner, and to observe that the activities with statistical graphics are presented in an equitable manner in relation to their demand and quantity.

Although this research declares novel and relevant information from the analysis of all the latest textbooks proposed for Chilean rural education, this study is limited to the description of the most relevant units of analysis, and future complementary research should use others for a more exhaustive analysis. Another limitation is that, although this study involves all Chilean rural education, the results are not extrapolable to urban education because they have different textbooks. Therefore, another project is to make a comparison between the formative and evaluative activities proposed in rural and urban schools to show differences and similarities.

## Figures and Tables

**Table 1:** Learning objectives for multigrade rural primary courses [33].

Grade	Objective
1st Grade	Collect and record data to answer statistical questions about oneself and the environment, using blocks, tally charts, and pictograms (p. 7). Construct, read, and interpret pictograms (p. 8).
2nd Grade	Collect and record data to answer statistical questions about games with coins and dice, using blocks, tally charts, and pictograms (p. 7). Record in tables and simple bar charts the results of random games with dice and coins (p. 8). Construct, read, and interpret pictograms with a scale and simple bar charts (p. 9).
3rd Grade	Conduct surveys, classify and organize the data obtained in tables, and display them in bar charts (p. 7). Represent data using dot plots (p. 8). Construct, read, and interpret pictograms and simple bar charts with a scale, based on collected or given information (p. 9).
4th Grade	Conduct surveys, analyze the data, and compare it with the results of random samples, using tables and graphs (p. 7). Perform random playful and everyday experiments, and tabulate and represent them through charts manually and/or with educational software (p. 8). Read and interpret pictograms and simple bar charts with a scale, and communicate findings (p. 9).
5th Grade	Read, interpret, and complete tables, simple bar charts, and line charts, and communicate findings (p. 7). Use stem-and-leaf plots to represent data from random samples (p. 9).
6th Grade	Read and interpret double bar and pie charts, and communicate findings (p. 7). Compare distributions of two groups from random samples, using dot plots and stem-and-leaf plots (p. 9).

**Table 2:** Descriptions of reading levels on a statistical graph [34,35,36].

Level	Description
Reading the data	Literal reading of the information, which is present in the statistical graph.
Reading between the data	Implicit reading of information, obtained from simple mathematical calculations or comparisons.
Reading beyond the data	Reading information that is not present in the statistical graph, involves predicting trends or values, taking into consideration the data shown in it (reasoning based on the information).
Reading behind the data	Critical evaluation is required of how the data are collected, how they are represented, and the conclusions drawn. It demands reflection on context and mathematical or statistical knowledge.

**Table 3:** Descriptions of levels of semiotic complexity in a statistical graph [7,37].

Level	Description
Representation of individual data	The graph displays isolated data points. Concepts of variable or distribution are not used.
Representation of a data set, without summarizing its distribution	Each data point of a distribution is shown. The concept of frequency or frequency distribution is not used, but the concept of variable is.
Representation of a data distribution	The data distribution is shown, which includes the calculation of frequencies.
Representation of multiple data distributions	Two or more frequency distributions are displayed.

**Table 4:** Coding of textbooks for multigrade education analyzed.

Code	Reference
T1	MINEDUC. Cuaderno de trabajo 1º Básico. ¡La aventura de aprender!: Unidades 5 y 6 Tiempo y Geometría Estadística. Matemática. Módulo didáctico para la enseñanza y aprendizaje en escuelas rurales multigrado; MINEDUC: Santiago, Chile, 2021. [MINEDUC. Workbook for 1st Grade. The Adventure of Learning: Units 5 and 6 Time and Statistical Geometry. Mathematics. Teaching and learning module for multigrade rural schools; MINEDUC: Santiago, Chile, 2021]
T2	MINEDUC. Cuaderno de trabajo 2º Básico. ¡La aventura de aprender!: Unidades 5 y 6 Tiempo y Geometría Estadística. Matemática. Módulo didáctico para la enseñanza y aprendizaje en escuelas rurales multigrado; MINEDUC: Santiago, Chile, 2021. [MINEDUC. Workbook for 2nd Grade. The Adventure of Learning: Units 5 and 6 Time and Statistical Geometry. Mathematics. Teaching and learning module for multigrade rural schools; MINEDUC, Santiago, Chile, 2021]
T3	MINEDUC. Cuaderno de trabajo 3º Básico. ¡La aventura de aprender!: Unidades 5 y 6 Tiempo y Geometría Estadística. Matemática. Módulo didáctico para la enseñanza y aprendizaje en escuelas rurales multigrado. MINEDUC: Santiago, Chile, 2021. [MINEDUC. Workbook for 3rd Grade. The Adventure of Learning: Units 5 and 6 Time and Statistical Geometry. Mathematics. Teaching and learning module for multigrade rural schools; MINEDUC: Santiago, Chile, 2021]
T4	MINEDUC. Cuaderno de trabajo 4º Básico. ¡La aventura de aprender!: Unidades 5 y 6 Tiempo y Geometría Estadística. Matemática. Módulo didáctico para la enseñanza y aprendizaje en escuelas rurales multigrado; MINEDUC: Santiago, Chile, 2021. [MINEDUC. Workbook for 4th Grade. The Adventure of Learning: Units 5 and 6 Time and Statistical Geometry. Mathematics. Teaching and learning module for multigrade rural schools; MINEDUC: Santiago, Chile, 2021]
T5	MINEDUC. Cuaderno de trabajo 5º Básico. ¡La aventura de aprender!: Unidades 5 y 6 Tiempo y Geometría Estadística. Matemática. Módulo didáctico para la enseñanza y aprendizaje en escuelas rurales multigrado; MINEDUC: Santiago, Chile, 2021. [MINEDUC. Workbook for 5th Grade. The Adventure of Learning: Units 5 and 6 Time and Statistical Geometry. Mathematics. Teaching and learning module for multigrade rural schools; MINEDUC: Santiago, Chile, 2021]
T6	MINEDUC. Cuaderno de trabajo 6º Básico. ¡La aventura de aprender!: Unidades 5 y 6 Tiempo y Geometría Estadística. Matemática. Módulo didáctico para la enseñanza y aprendizaje en escuelas rurales multigrado; MINEDUC: Santiago, Chile, 2021. [MINEDUC. Workbook for 6th Grade. The Adventure of Learning: Units 5 and 6 Time and Statistical Geometry. Mathematics. Teaching and learning module for multigrade rural schools; MINEDUC: Santiago, Chile, 2021]

**Table 5:** Distribution of activities with statistical graphs in textbooks for rural multigrade education in primary school.

Course	Formation Activities (n = 82)	Assessment Activities (n = 20)	Total (n = 102)
1	14 (17.1)	2 (10)	16 (15.7)
2	11 (13.4)	3 (15)	14 (13.7)
3	10 (12.2)	4 (20)	14 (13.7)
4	7 (8.5)	4 (20)	11 (10.8)
5	15 (18.3)	2 (10)	17 (16.7)
6	25 (31.7)	5 (25)	30 (29.4)
Total	82 (100)	20 (100)	102 (100)

**Table 6:** Distribution of types of statistical graphs present in activities in textbooks for rural multigrade education in primary.

Type of Graph	1st (n = 16)		2nd (n = 16)		3rd (n = 14)		4th (n = 11)		5th (n = 18)		6th (n = 31)		Total (n = 106)	
	FA	AA	FA	AA	FA	AA	FA	AA	FA	AA	FA	AA	FA	AA
Pictogram	14 (100)	2 (100)	6 (46.2)	1 (33.3)	1 (10)	1 (25)		1 (25)					21 (24.4)	5 (25)
Bars			7 (53.9)	2 (66.7)	6 (60)	1 (25)	7 (100)	3 (75)	2 (12.6)		8 (30.8)	2 (40)	30 (34.9)	8 (40)
Points					3 (30)	2 (50)			4 (25)		5 (19.3)	1 (20)	12 (14)	3 (15)
Line									3 (18.8)	1 (50)			3 (3.5)	1 (5)
Stem and leaf									5 (31.3)	1 (50)	7 (26.9)	1 (20)	12 (14)	2 (10)
Pie											6 (23.1)	1 (20)	6 (7)	1 (5)
Choice									2 (12.6)				2 (2.3)	
Total	14 (100)	2 (100)	13 (100)	3 (100)	10 (100)	4 (100)	7 (100)	4 (100)	16 (100)	2 (100)	26 (100)	5 (100)	86 (100)	20 (100)

Note. FA: Formation activities; AA: Assessment activities.

**Table 7:** Distribution of reading level present in activities with statistical graphs in textbooks for rural multigrade education in primary education.

Reading Level	1st (n = 16)		2nd (n = 14)		3rd (n = 14)		4th (n = 11)		5th (n = 17)		6th (n = 30)		Total (n = 102)	
	FA	AA	FA	AA	FA	AA	FA	AA	FA	AA	FA	AA	FA	AA
1	1 (7.1)	1 (50)	2 (18.2)	1 (33.3)	2 (20)	1 (25)		1 (25)	1 (6.7)				6 (7.3)	4 (20)
2	3 (21.4)	1 (50)	5 (45.5)	2 (66.7)		3 (75)	2 (28.6)	3 (75)	2 (13.3)	2 (100)	8 (32)	5 (100)	20 (24.4)	16 (80)
4	10 (71.4)		4 (36.4)		8 (80)		5 (71.4)		12 (80)		17 (68)		56 (68.3)	
Total	14 (100)	2 (100)	11 (100)	3 (100)	10 (100)	4 (100)	7 (100)	4 (100)	15 (100)	2 (100)	25 (100)	5 (100)	82 (100)	20 (100)

Note. FA: Formation activities; AA: Assessment activities.

**Table 8:** Distribution of level of semiotic complexity present in activities with statistical graphs in textbooks for rural multigrade education in primary education.

Semiotic Level	1st (n = 16)		2nd (n = 14)		3rd (n = 14)		4th (n = 11)		5th (n = 17)		6th (n = 31)		Total (n = 103)	
	FA	AA	FA	AA	FA	AA	FA	AA	FA	AA	FA	AA	FA	AA
2	1 (7.1)				1 (10)				5 (33.3)	1 (50)			7 (8.4)	1 (5)
3	13 (92.9)	2 (100)	11 (100)	3 (100)	9 (90)	4 (100)	7 (100)	4 (100)	10 (66.7)	1 (50)	16 (61.5)	2 (40)	66 (79.5)	16 (80)
4											10 (38.5)	3 (60)	10 (12.1)	3 (15)
Total	14 (100)	2 (100)	11 (100)	3 (100)	10 (100)	4 (100)	7 (100)	4 (100)	15 (100)	2 (100)	26 (100)	5 (100)	83 (100)	20 (100)

Note. FA: Formation activities; AA: Assessment activities.

**Table 9:** Distribution of type of tasks present in activities with statistical graphs in textbooks for rural multigrade education in primary education.

Task	1st (n = 16)		2nd (n = 14)		3rd (n = 14)		4th (n = 11)		5th (n = 17)		6th (n = 31)		Total (n = 102)	
	FA	AA	FA	AA	FA	AA	FA	AA	FA	AA	FA	AA	FA	AA
Read	4 (28.7)	1 (50)	8 (72.3)	2 (66.7)		1 (25)		1 (25)	6 (40)		10 (38.5)	2 (40)	28 (33.7)	7 (35)
Calculate	9 (64.3)	1 (50)	8 (72.3)	3 (100)	5 (50)	3 (75)	3 (42.9)	3 (75)	10 (66.7)	2 (100)	20 (76.9)	5 (100)	55 (66.3)	17 (85)
Complete	3 (21.4)	1 (50)	2 (18.1)	1 (33.3)	3 (30)	1 (25)	1 (14.3)		4 (26.7)		3 (11.5)		17 (20.5)	3 (15)
Build	2 (14.3)		2 (18.1)		5 (50)		3 (42.9)	1 (25)	3 (20)		2 (7.7)		17 (20.5)	1 (5)
Justify	8 (57.1)		4 (36.4)		7 (70)		3 (42.9)		11 (73.3)		15 (57.7)		48 (57.8)	
Create Question	2 (14.3)		3 (27.3)		1 (10)		2 (28.6)		2 (13.3)		3 (11.5)		13 (15.7)	
Compare							1 (14.3)						1 (1.2)	
Fill in table											1 (3,9)		1 (1.2)	
Total	14 (100)	2 (100)	11 (100)	3 (100)	10 (100)	4 (100)	7 (100)	4 (100)	15 (100)	2 (100)	26 (100)	5 (100)	83 (100)	20 (100)

Note. FF: Formation activities; AA: Assessment activities.

**Table 10:** Distribution of type of context present in activities with statistical graphs in textbooks for rural multigrade education in primary education.

Context	1st (n = 16)		2nd (n = 14)		3rd (n = 14)		4th (n = 11)		5th (n = 17)		6th (n = 31)		Total (n = 102)	
	FA	AA	FA	AA	FA	AA	FA	AA	FA	AA	FA	AA	FA	AA
Personal	12 (85.7)	2 (100)	10 (90.9)	3 (100)	6 (60)	3 (75)	3 (42.9)	3 (75)	6 (40)		16 (61.5)	4 (80)	51 (61.5)	15 (75)
Work	1 (7.1)				4 (40)	1 (25)	4 (57.1)	1 (25)	7 (46.7)	1 (50)	9 (34.6)	1 (20)	25 (30.1)	4 (20)
Social	1 (7.1)		1 (9.1)										2 (2.4)	
Scientific									2 (13.3)	1 (50)	1 (3.9)		3 (3.6)	1 (5)
Total	14 (100)	2 (100)	11 (100)	3 (100)	10 (100)	4 (100)	7 (100)	4 (100)	15 (100)	2 (100)	26 (100)	5 (100)	83 (100)	20 (100)

Note. FA: Formation activities; AA: Assessment activities.

## Data Availability

Data supporting the results of the present study will be made available by the authors upon reasonable request.
